# Impact of ethnic-specific guidelines for anti-hypertensive prescribing in primary care in England: a longitudinal study

**DOI:** 10.1186/1472-6963-14-87

**Published:** 2014-02-25

**Authors:** Lena Barrera, Craig Leaper, Utz J Pape, Azeem Majeed, Marta Blangiardo, Christopher Millett

**Affiliations:** 1 Department of Primary Care and Public Health, School of Public Health, Imperial College London, London W6 8RP, UK; 2 Department of Internal Medicine, Faculty of Health, Universidad del Valle, Cali, Colombia; 3 Department of Epidemiology and Biostatistics, School of Public Health, Imperial College London, London W2 1NY, UK; 4 Department of Primary Care and Public Health, School of Public Health, Imperial College London, Charing Cross Campus, 3rd Floor, Reynolds Building, St Dunstan's Road, London W6 8RP, UK

**Keywords:** Hypertension, Hypertension guidelines, Primary care, Antihypertensive drugs, Trend analysis, Antihypertensive prescribing

## Abstract

**Background:**

In England, the National Institute for Health and Care Excellence (NICE) produces guidelines for the management of hypertension. In 2006, the NICE guidelines introduced an ethnic-age group algorithm based on the 2004 British Hypertension Society guidelines to guide antihypertensive drug prescription.

**Methods:**

A longitudinal retrospective study with 15933 hypertensive patients aged 18 years or over and registered with 28 general practices in Wandsworth, London in 2007 was conducted to assess variations in antihypertensive prescribing. Logistic models were used to measure variations in the odds of being prescribed the 2006 NICE first line recommended monotherapy among NICE patient groups over the period.

**Results:**

From 2000 to 2007, the percentage of patients prescribed the recommended monotherapy increased from 54.2% to 61.4% (p < 0.0001 for annual trend). Over the study period, black patients were more likely to be prescribed the recommended monotherapy than younger non-black patients (OR 0.16, 95% CI 0.12 – 0.21) and older non-black patients (OR 0.49, 95% CI 0.37 – 0.65). After the introduction of the NICE guidelines there was an increase in the NICE recommended monotherapy (OR 1.44, 95% CI 1.19 – 1.75) compared with the underlying trend. Compared to black patients, an increase in the use of recommended monotherapy was observed in younger non-black patients (OR 1.49, 95% CI 1.17 – 1.91) but not in older non-black patients (OR 0.58, 95% CI 0.46 – 0.74).

**Conclusion:**

The introduction of the 2006 NICE guideline had the greatest impact on prescribing for younger non-black patients. Lower associated increases among black patients may be due to their higher levels of recommended prescribing at baseline. The analysis suggests that guidelines did not impact equally on all patient groups.

## Background

Observational studies have consistently shown that fewer than 40% of hypertensive patients worldwide have controlled blood pressure [1]. Hypertensive patients either without treatment or with inappropriate therapy are an important cause of the current low rates of blood pressure control [1,2]. Lack of treatment intensification or ‘clinical inertia’ has been recognized as a frequent cause of uncontrolled blood pressure in hypertensive patients managed in primary care [3]. Clinical guidelines are produced, and frequently updated, to guide physicians on choosing optimal antihypertensive therapy as a strategy to reduce clinical inertia [4,5].

Physician criteria for antihypertensive prescribing may differ from that established in clinical guidelines. Surveys have revealed sub-optimal use of clinical guidelines by physicians for prescribing antihypertensive drug therapy [6-15]. This may be due to concerns among physicians that the clinical guideline recommendations may not apply to all hypertensive patients in primary care [12,16-19]. Variations in hypertension treatment across primary care practices have additionally documented the gap between guidelines and physician antihypertensive drug preferences [20].

In England, the National Institute for Health and Care Excellence (NICE) is the authoritative body for the production of clinical guidelines [4]. The NICE guidelines are also used as reference to measure the performance of primary care services [21]. Until the last NICE hypertension guidelines published in 2011 [4], the management of hypertension in England primary care was based on the recommendations established in the 2004 NICE hypertension guidelines [22]. In 2006, an NICE guidance update was introduced advising that those patients of black ethnicity and those aged 55 years or over should be treated differently to the rest of the population in terms of the first line antihypertensive monotherapy. Between January and April NICE extensively promoted that guideline and it was finally adopted in June 2006.

In England increases in antihypertensive prescribing for hypertensive patients have been documented; however the use of antihypertensive treatments established in the guidelines has been less explored. Because the antihypertensive treatment established in the 2006 updated NICE guidelines was based on the 2004 BHS (British Hypertension Society) guidelines and studies already published, these recommendations may have already been used before the introduction of that guideline. To assess the use of the NICE hypertension guideline recommendations by general practitioners, we examined variations in drug antihypertensive prescribing among hypertensive patients managed in England primary care over a 10 years follow-up period. The recommended treatment established in the 2006 updated NICE guidelines was used as the reference guideline. The impact of the introduction of this guideline on antihypertensive prescribing was also assessed.

## Methods

The data used for this study was derived from 28 general practices located in Wandsworth, Southwest London. We identified all patients with essential hypertension that had a clinical record in 2007 using diagnostic READ codes in computerized general practice records. READ codes are the clinical classification system used in primary care in the United Kingdom (UK). For each patient, we retrospectively extracted data on annual antihypertensive prescribing and clinical variables registered between 2000 and 2007. The study was part of a research programme that received ethics approval from the Wandsworth Local Research Ethics Committee.

We only included patients without missing or unknown record of race/ethnicity and aged 18 years or over during the study period. Patients who were registered for the first time in the respective year were counted as newly hypertensive patients. Data on whether the patient was prescribed the following antihypertensive drug class: angiotensin converting enzyme inhibitors (ACEI), beta-blockers (BB), calcium antagonist blockers (CCB), and diuretics (DD) was extracted annually. We defined a patient with an additional cardiovascular comorbidity as one who had one or more of the following diseases: diabetes mellitus, coronary heart disease, stroke, atrial fibrillation, renal failure and heart failure. Race/ethnicity was based on the information provided by the patient and registered according to that defined in the 2001 UK census [23].

We used the 2006 NICE guidelines as a reference guideline to assess variations in antihypertensive prescribing over the study period. The guideline was reviewed in January 2006 and the updated version was launched in June 2006. Because that guideline established the recommendations on the first line antihypertensive monotherapy based on a patient’s age and race/ethnicity (Table 1), we stratified our hypertensive population such as: b*lack* for patients of black ethnicity at all ages, *younger non-black* for patients of other ethnic origin different from black aged below 55 years and *older* non-black for patients of other ethnic origin different from *black* aged 55 years or over. Therefore, the older non-black and younger non-black patient groups include those from white, South Asian, Other Asian and other ethnic origins [24,25].

**Table 1:** First line monotherapy treatment established in the 2006 NICE hypertension guidelines

**NICE group**	**Younger than 55 years**	**55 years older or black at any age**
First line monotherapy treatment	Angiotensin coverting enzyme inhibitors (ACEI)	Calcium channels blockers (CCB) or Diuretics (D)

To examine variations in antihypertensive prescribing over the study period, we measured the following annual percentages a) the percentage of patients on antihypertensive treatment as the number of patients being prescribed no antihypertensive drug, one antihypertensive drug and two or more antihypertensive drugs over the total number of patients in each NICE patient group; b) the percentage of patients on ACEI, BB, CCB and DD monotherapy as number of patients prescribed each antihypertensive drug class over the number of patients on monotherapy in each NICE patient group; c) the annual percentage of patients on the recommended monotherapy as the number of patients prescribed the recommended monotherapy over the total number of patients on monotherapy in each NICE patient group. Because the recommendations established in the 2006 NICE guidelines applied to hypertensive patients aged 18 years or over without other cardiovascular comorbidity, we included only this hypertensive patient category to calculate the last two percentages [24,25].

### Statistical analysis

Patient characteristics are described as means and percentages. Unadjusted trend analysis for the percentage of hypertensive patients being prescribed antihypertensive treatment, the percentage of those on antihypertensive drug class and the percentage of those on the first line 2006 NICE recommended monotherapy between 2000 and 2007 was performed using the Stata test for trend assessment [26].

We used generalized estimating equations (GEE) with logit function to estimate variations in the odds ratio of being prescribed ACEI, BB, CCB and DD as monotherapy throughout the study period. We chose GEE models to take into account the potential correlation among the multiple drug prescriptions received by each patient over the study period and so obtaining more accurate estimators [27,28]. Additionally, because the GEE models measure the average variation in prescribing across population groups [27], we ran a model for each NICE patient group in each drug antihypertensive class. For these models, we firstly tested the annual variation in the odds ratio including a calendar year as a continuous variable. In order to examine the influence of the national guidelines in the prescription of each drug class, we added the variables for the introduction of both the 2004 BHS guidelines and the 2006 NICE guidelines. The effect of the 2006 NICE guidelines on each drug-related use was measured by including a dummy variable representing the before (2000-2005 years) and after (2006-2007 years) periods of the introduction of this guideline. The assessment of the introduction of the BHS guidelines was performed by using a dummy variable where the before period was between 2000 and 2003 and the after period from 2004 to 2007 [27]. The models included all hypertensive patients registered without additional cardiovascular comorbidity. A separated model for those newly registered was not performed due to the small samples in some NICE patient groups. The independence model criterion (QIC) was used to select the best suitable correlation structure for each model [29]. A robust estimation for the standard errors was performed. The model was formulated as follows:

$$Y=\beta *t+\beta_{і}*\mathrm{BHS}+\beta_{2}\;*\mathrm{NICE}\;$$

The *β* * *t* term was used to adjust for the baseline trend in prescribing so that changes in prescribing associated with the introduction of guidelines could be attributed to this intervention. The *β*_*і*_ * *BHS* term stands for the introduction of BHS guidelines and the  *β*_2_  * *NICE* term stands for the introduction of NICE guidelines.

To assess the variation in the odds ratio of being prescribed the 2006 NICE first line recommended monotherapy treatment over the period, a logistic regression model with standard errors adjusted for practice cluster [30] for all registered patients and newly registered patients were performed respectively. For these models, the outcome was being prescribed the first line recommended monotherapy as a binary variable. The time variation of the outcome was measured by including year as continuous variable. The effect of the introduction of the NICE and BHS guidelines was assessed using dummy variables as described above. To evaluate whether or not the effect of the introduction of the NICE guidelines varied across the NICE patient groups, we included a term for interaction effect. Finally the models were adjusted for sex.

The data was analysed using STATA version 11 (Stata Corporation, College Station, TX, USA).

## Results

15933 hypertensive patients with a valid ethnicity code were selected from the registers of the participating practices in 2007. Among them, 9085 (57.0%) were white, 3926 (24.6%) were black, 1556 (9.8%) were South Asian, 594 (3.7%) were other Asian and 772 (4.8%) belonged to other ethnic groups. 9261 (58.1%) were over 55 years, 8755 (55%) were female and 6042 (37.90%) had at least one cardiovascular comorbidity. The most frequently associated comorbidity was diabetes, which was present in 3419 (21.5%) of patients, followed by coronary heart disease in 1842 (11.6%), stroke in 1145 (7.2%), atrial fibrillation in 756 (4.7%), renal failure in 670 (4.2%) and heart failure in 395 (2.5%). 4078 (44.3%) of older non-black patients had at least one cardiovascular comorbidity compared to 555 (20.2%) of younger non-black patients (Table 2).

**Table 2:** Characteristics of included patients, 2007

	**Black patients**	Younger non-black patients*	0lder non-black patients**	**Total**
Number of Patients number (%)	3926 (24.6)	2746 (17.2)	9261 (58.1)	15933
Mean age years (sd)	60.4 (13.2)	44.7 (7.8)	71.1 (9.9)	63.9 (14.5)
Male no (%)	1588 (40.5)	1493 (54.3)	4096 (44.2)	7177 (45.1)
Ethnicity different from Black number (%)				
White		1936 (70.5)	7149 (77.2)	9085 (75.7)
South Asian		403 (14.7)	1153 (12.5)	1566 (12.9)
Other Asian		160 (5.8)	434 (4.7)	594 (4.9)
Other origin		247 (8.9)	525 (5.7)	772 (6.4)
Presence of cadiovascular comorbidity				
No	2517 (64.1)	2191 (79.8)	5183 (55.9)	9891 (62.1)
Yes^***^	1409 (35.8)	555 (20.2)	4078 (44.3)	6042 (37.9)

% Indicate percentage.

sd Standard deviation.

*Indicate those of White, Asian, Other Asian origin or other ethnic origin <55 years.

**Included those of White, Asian, Other Asian origin or other ethnic origin aged 55 years and over.

***Patients with one or more than of the follow diseases: coronary heart diseases, diabetes mellitus, heart failure, atrial fibrillation stroke or renal failure.

### Use of antihypertensive treatment by number of drugs prescribed over the period

From 2000 to 2007, the percentage of patients not prescribed antihypertensive drug treatment decreased in all NICE patient groups. The percentage decreased from 43.2% to 30.2%, from 30.6% to 13.5% and from 25.2% to 8.9% in younger non-black patients, black patients and older non-black patients respectively (p < 0.0001, for annual trend for all). The percentage of newly registered patients on monotherapy prescription increased from 35.2% to 45.9%, from 38.6% to 48.6% in younger non-black patients and older non-black patients (p < 0.0001, for annual trend for all) respectively and from 33.7% to 45.2% for black patients (p = 0.0002) (Table 3).

**Table 3:** Variation in percentages of hypertensive patients on antihypertensive treatment between 2000 and 2007

	**2000**		**2007**		**p value for trend**	
	Newly registered*	**All patients**	**Newly registered**	**All patients**	**Newly registered**	**All patients**
Younger non black** number (%)‡						
No drug therapy	132 (43.8)	653 (43.2)	101 (28.1)	835 (30.2)	<0.0001	<0.0001
One antihypertensive drug	106 (35.2)	505 (33.4)	165 (45.9)	897 (32.7)	<0 0001	0.4106
Two or more antihypertensive drugs	63 (20.9)	353 (23.4)	93 (25.9)	1014 (36.9)	0.0623	<0.0001
**Total**	**301**	**1511**	**359**	**2748**		
**Black number (%)**						
No drug therapy	107 (34.6)	578 (30.6)	41 (17.9)	531 (13.5)	<0.0001	<0.0001
One antihypertensive drug	104 (33.7)	590 (30.7)	103 (45.2)	1115 (28.4)	0.0002	0.0877
Two or more antihypertensive drugs	98 (31.7)	755 (39.3)	84 (36.8)	2280 (58.1)	0.5019	<0 0001
**Total**	**309**	**1923**	**228**	**3926**		
Older non-black*** number (%)						
No drug therapy	176 (28.9)	1012 (25.2)	49 (11.9)	831 (8.9)	<0.0001	<0.0001
One antihypertensive drug	235 (38.6)	1490 (37.1)	199 (48.6)	2854 (30.8)	<0.0001	<0.0001
Two or more antihypertensive drugs	198 (32.5)	1519 (37.8)	162 (39.5)	5576 (60.2)	0.0001	<0.0001
**Total**	**609**	**4021**	**410**	**9261**		
**Total**	**1219**	**7455**	**997**	**15933**		

*Patients first time registered with hypertension diagnosis over the referred year.

**Included those of White, Asian, Other Asian and other ethnic origin aged <55 years.

***Included those of White, Asian, Other Asian and other ethnic origin aged >=55 years.

‡Percentage = number of patients in each drug category/ Total patient-group in each year*100.

### Use of antihypertensive drug class in hypertensive patients without cardiovascular comorbidity over the period

#### ACEI

Between 2000 and 2007, the percentage of patients on ACEI monotherapy changed as follows: in younger non-black patients from 26.4% to 53.7% (p < 0.0001 for annual trend), in older non-black patients from 18.9% to 33.4% (p < 0.0001 for annual trend) and in black patients from 9.2% to 11.7% (p = 0.260 for annual trend).

**Figure 1:**
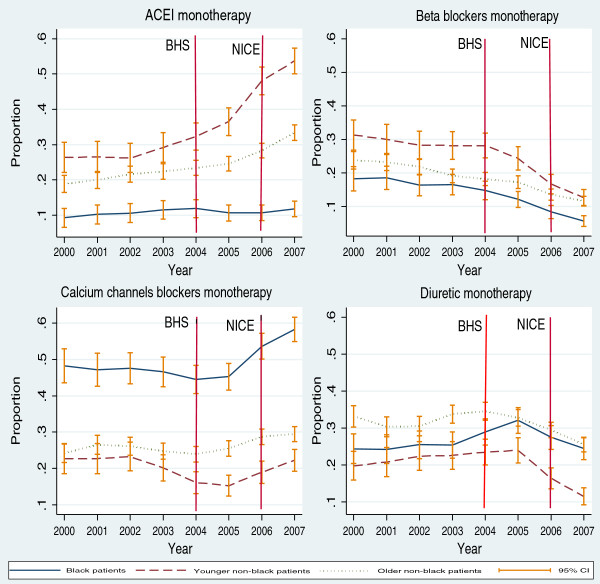
Mean annual proportion of hypertensive patients without cardiovascular comorbidity* on drug-related monotherapy by NICE patient groups. Percentage: number of patients on monotherapy/total of patients on monotherapy in each patient group by drug classes. Vertical line indicated the introduction year of the BHS and NICE guidelines. *Patients without one of the follow diseases coronary heart disease, diabetes mellitus, heart failure, atrial fibrillation, stoke or renal failure.

The annual mean proportion of hypertensive patients without comorbidity on ACEI monotherapy estimated from GEE models is shown in Figure 1. The prescription of ACEI was higher for younger non-black patients compared to other NICE patient groups over the period. The models also revealed that for this patient group there was an additional increase of 23% in the odds of being prescribed ACEI as monotherapy (OR 1.23 95% CI 1.10 – 1.38) after the introduction of the 2006 NICE guideline. However, a significant variation in the use of ACEI after the introduction of the 2006 NICE guidelines was not observed for black patients and older non-black patients, (OR 0.99 95% CI 0.88 – 1.13) and (OR 1.06 95% CI 0.99 – 1.12) respectively (Figure 1). See appendix for GEE models (Additional file 1).

### Beta blockers

During 2000-2007, the percentage of patients prescribed BB monotherapy decreased from 31.3% to 12.6%, (p < 0.0001 for annual trend) in younger non-black patients; from 23.8% to 11.6% in older non-black patients, (p < 0.0001 for annual trend) and from 18.2% to 5.6%, (p < 0.0001) for annual trend) in black patients.

From the GEE models, a reduction in the mean annual proportion of patients prescribed BB as monotherapy was also observed for all NICE patient groups over the period (Figure 1). This decline accelerated after the introduction of the 2006 NICE guidelines. Thus, the odds of being prescribed BB for all NICE patient groups were as follows: for black patients (OR 0.57 95% CI 0.45 – 0.74), for younger non-black patients (OR 0.57 95% CI 0.46 – 0.71) and for older non-black (OR 0.91 95% CI 0.85 – 0.98) (Figure 1). See appendix for GEE models (Additional file 1).

### Calcium channel blockers

There was an increase in the percentage of black patients on CCB monotherapy from 48.2% in 2000 to 58.3% in 2007 (p < 0.0001 for annual trend). The percentage stayed constant from 22.7% in 2000 and 22.4% in 2007 in younger non-black (p = 0.102 for annual trend) and increased from 24.4% to 29.5% (p = 0.0008 for annual trend) in older non-black patients.

From the GEE models, the highest mean annual proportion of hypertensive patients on CCB monotherapy was observed in black patients over the study period. The proportion steeply increased after 2005 and picked up at 2007 (Figure 1). After the introduction of the 2006 NICE guidelines, an increase of 22% in the odds of being prescribed CCB as monotherapy was also observed in this patient group, (OR 1.22 95% CI 1.10 – 1.34). By contrast, there was no a significant variation in the odds of being on CCB monotherapy for younger non-black patients and older non-black patients, (OR 1.14 95% CI 0.99 – 1.31) and (OR 1.05 95% CI 0.99 – 1.12) respectively (Figure 1). See appendix for GEE models (Additional file 1).

### Diuretics

The percentage of black patients on DD monotherapy was 24.3% in 2000 and 24.0% in 2007 (p = 0.2046 for annual trend). There were fluctuations in this prescribing DD pattern over the period with an increase in the percentage in 2005. Between 2000 and 2007 the percentage changed from 19.1% to 11.0% (p = <0.0001 for annual trend) in younger non-black patients and from 33.1% to 25.3% (p = 0.0001 for annual trend) in older non-black patients.

The mean annual proportion of DD therapy estimated from GEE models started decreasing after 2005 in all NICE patient groups (Figure 1). The introduction of 2006 NICE guidelines was associated with an additional reduction in the odds of DD monotherapy use in all NICE patient groups such as for black patients (OR 0.77 95% CI 0.69 – 0.86), for younger non-black patients (OR 0.71 95% CI 0.61 – 0.81) and for older non-black patients (OR 0.77 95% CI 0.69 – 0.86) (Figure 1). See appendix for GEE models (Additional file 1).

### Use of the 2006 first line NICE recommended monotherapy over the period

#### All patients without cardiovascular comorbidity

**Figure 2:**
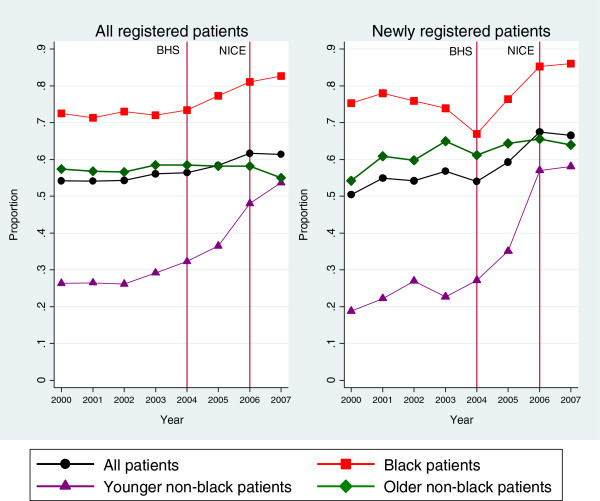
Variation in percentage of hypertensive patients* on recommended monotherapy. Percentage: patients on monotherapy/total patients on monotherapy by each NICE patients group *100. *Patients without any the follow diseases coronary heart disease, diabetes mellitus, heart failure, atrial fibrillation, stroke or renal failure.

Between 2000 and 2007, the percentage of patients prescribed with recommended monotherapy increased from 54.2% to 61.4% (p < 0.0001 for annual trend). For black patients, there was a change from 72.5% to 82.6% (p = 0.0001 for annual trend), for younger non-black patients from 26.4% to 53.7% (p < 0.0001 for annual trend) and for older non-black patients from 57.4% to 55.0% (p = 0.765 for annual trend) (Figure 2). The logistic model showed that overall black patients were significantly more likely to be prescribed the first line recommended monotherapy than younger non-black (OR 0.16 95% CI 0.12 – 0.21) and older non-black patients (OR 0.49 95% CI 0.37 – 0.65) respectively over the study period. After the introduction of the guidelines (period 2006 – 2007), there was an increase in the use of the first line NICE recommended monotherapy (OR 1.44 95% CI 1.19 – 1.75) compared with the period before (2000 – 2005). Compared to black patients, younger non-black patients were more likely to be prescribed the NICE recommended monotherapy (OR 1.49 95% CI 1.17 – 1.91) but older patients were less likely to be prescribed the NICE recommended treatment (OR 0.58 95% CI 0.46 – 0.74) (Table 4) (Figure 2).

**Table 4:** **Variation in the odds ratio of being prescribed the first line NICE recommended monotherapy**_**₁ **_among hypertensive patients without cardiovascular comorbidity*

	**All hypertensive patients**			**Newly registered hypertensive patients**		
**Variable**	**Odds ratio**	**95% CI‡**	**P value**	**Odds ratio**	**95% CI**	**P value**
NICE^2^	1.44	1.19–1.75	<0.0001	1.62	1.10–2.36	0.013
References period between 2000–2005						
Interaction term NICE* guidelines groups						
Younger non–black patients* NICE	1.49	1.17–1.91	0.001	1.83	1.17–2.87	0,008
Older non black patients* NICE	0.58	0.46–0.74	<0.0001	0.56	0.39–0.77	0.001
Reference						
Black patients* period between 2000–2005						
NICE patients groups						
Younger non–black patients	0.16	0.12–0.21	<0.0001	0.12	0.07–0.19	<0.0001
Older non–black patients	0.49	0.37–0.65	<0.0001	0.55	0.39–0.77	0.001
Reference non–black patients						
Year	1.02	0.98–1.05	0.247	1.10	1.01–1.20	0.037
Sex	0.89	0.79–1.00	0.079	1.00	0.84–1.19	0.977
Reference female						
BHS^3^	1.06	0.98–1.15	0.119	0.85	0.65–1.08	0.190
Reference period between 2000 and 2003						

^1^Based on the 2006 NICE hypertension guideline update reference 24.

*Patients without one of the follow disease: coronary heart disease, diabetes mellitus, heart failure, atrial fibrillation, disease, stroke or renal failure.

‡CI confidence interval.

^2^NICE a variable which stands for the period after introduction of the 2006 NICE guidelines 2006–2007.

^3^BHS a variable which stands for the period after the introduction of the 2004 BHS guidelines 2004–2007.

#### Newly registered patients without cardiovascular comorbidity

From 2000 to 2007, the percentage of newly registered patients on the first line recommended monotherapy increased from 50.5% to 66.6% (p < 0.0001 for annual trend). For black patients, the percentage increased from 75.3% to 86.1% (p < 0.065 for annual trend), for younger patients from 18.8% to 58.1% (p < 0.0001 for annual trend) and for older patients from 54.3% to 63.9% (p = 0.0442 for annual trend) (Figure 2). The logistic model showed that over the study period, younger non-black patients and older patients non-black were less likely to have the first line recommended monotherapy (OR 0.12 95% CI 0.07 – 0.19) and (OR 0.55 95% CI 0.39 – 0.77) respectively compared to black patients. Compared to period between 2000 and 2005, a significant increase in the use of the first line recommended monotherapy was quantified in the period between 2006 and 2007, overall (OR 1.62 95% CI 1.10 – 2.36). After the introduction of the 2006 NICE guidelines, compared to black patients, younger non-black patients were more likely to be prescribed the first line recommended monotherapy (OR 1.83, 95% CI 1.17 – 2.87) but older non-black patients were less likely to be prescribed with the first line recommended monotherapy (OR 0.56 CI 95% 0.39 – 0.77) (Table 4) (Figure 2).

## Discussion

There have been major changes in antihypertensive prescribing between 2000 and 2007 in this UK primary care setting. Over this period, the main changes were an annual decrease in the percentage of all hypertensive patients with no antihypertensive medication. The recommended monotherapy treatment established in the 2006 NICE guidelines for patients of black origin started using before the introduction of this guideline. Black patients were additionally more likely to be prescribed CCB monotherapy after the introduction of 2006 NICE guideline. ACEI was progressively incorporated as antihypertensive monotherapy for younger non-black patients through the study period. The introduction of the 2006 NICE guidelines was associated with an additional increase in the use of ACEI monotherapy in this patient group. There was a slight variation in the monotherapy prescribing pattern used for older non-black patients over the study period. Our analysis suggests that guidelines did not impact equally on all patient groups.

On overall 61.4% of hypertensive patients were on the 2006 NICE first line monotherapy antihypertensive treatment at the end of the study period. The use of this recommended monotherapy seems to start before the introduction of these guidelines particularly in those of black origin. Evidence from clinical trials published before the introduction of these guidelines showing a differential antihypertensive response to CCB in patients of black origin [31] may influenced the early use of this antihypertensive treatment. Although the use of ACEI in younger non-black constantly increased over the period, the introduction of the guideline clearly reinforced its prescribing among general practitioners. Thus, after the implementation of the guidelines younger non-black patients experienced a nearly 50% increase in the odds of being prescribed the recommended monotherapy compared to black patients. Our findings on the use of antihypertensive treatment recommended in the national guidelines look similar to that have been reported. In 2003, Walley et al. reported that 54% of hypertensive patients were on the first line antihypertensive treatment established in the former BHS hypertension guidelines [32]. More recently, in a cohort of patients with stroke, Toshcke et al. found that the use of the antihypertensive recommended treatment established in the 2004 BHS guidelines increased from 24% to 37% between 1997 and 2006 [33]. In a cross-sectional study of 51 general practices located in Lambeth, London, Schofield et al. also found that 44% of hypertensive patients were prescribed the 2006 guidelines recommended antihypertensive treatment [34]. In addition to this overall use, our results remarked the influence of the guidelines on the first line prescribing particularly in younger non-black patients.

The 2006 NICE guidelines recommended that either a CCB or DD should be used as the first line monotherapy in older non-black patients [24], but on overall the percentage of older patients on either CCB or DD slightly decrease from 57.4% to 55.0% over the study period. Moreover, compare to black patients, the odds of being on that recommended monotherapy treatment was significantly lower in older non-black patients (OR 0.49 CI 95% 0.37 – 0.65) and this prescribing pattern did not improve after the introduction of the guidelines. This result differ from that published by NICE in 2010 where an increase in CCB prescribing for newly hypertensive patients aged 55 years and older was found. However, the report did not examine differences between black patients and those of other ethnic origin [35]. Schofield et al. also found that among all 2006 NICE patient groups, older non-black patients had the lowest percentage of those being prescribed the 2006 NICE recommended treatment [34]. Elderly hypertensive patients may be treated differently in routine clinical settings. For these patients the use of higher blood pressure targets and the prescription of few antihypertensive drugs for the treatment of hypertension have been observed in different surveys. This is despite guideline recommendations state that hypertension should be actively managed in older persons [36,37]. In general practice a low use of DD in elderly hypertensive patients has also been reported [7]. The divergence between the guideline recommendations and the antihypertensive treatment offered to older non-black patients observed in our study may also be due to the effect of other non-cardiovascular comorbidities, side effects or individual physician preferences influencing choice of antihypertensive drug made by general practitioners [7].

The highest percentage of hypertensive patients on CCB monotherapy was in black patients. Moreover the introduction of the 2006 NICE guidelines was associated with an additional increase in the use of CCB for this NICE patient group. They were also more likely to be on the recommended monotherapy (CCB or DD) compared to younger non-black patients and older non-black patients over the study period. In 2005, we had already reported this prescribing pattern in black patients [38]. Similarly, Schofield et al. reported that nearly 90% of black patients were prescribed the 2006 NICE recommended antihypertensive treatment among hypertensive patients treated in primary care [34]. A differential antihypertensive treatment provided to black patients has also been documented elsewhere. In a trend analysis of the National Health and Nutrition Examination Surveys (NHANES) between 2001 and 2010, Gu et al. found that patients of black origin not only were more likely to be prescribed CCB or DD but also the percentage of black patients on CCB or DD therapy increased over the period [39]. The recommendations established in the Joint National Committee on Prevention, Detection, Evaluation and Treatment of High Blood Pressure (JNC 7) may have influenced these trends. Hence, it seems that the recommendations on antihypertensive treatment for black patients established in national guidelines have been highly adopted in clinical practice.

For all NICE patient groups there has been a significant reduction in the use of beta blockers as monotherapy antihypertensive treatment. This reduction was significantly intensified by the introduction of the NICE guidelines. Similarly, the 2010 NICE implementation uptake report showed a decline in the percentage of BB usage related to all hypertensive drugs, from nearly 25% in February 2006 to 21% in October 2009. The decline was time-related to the introduction of the 2006 NICE guidelines [35]. In addition to guidelines, general practitioners aware of some evidence that beta blockers may be less effective at reducing blood pressure [40], as well as of their association with new onset diabetes [41] may contribute to the constant reduction in BB use over the period. However opposite trends in BB prescribing have also been observed. Using NHANES data, Gu et al. found an increase in the use BB either as monotherapy or polytherapy between 1998 and 2010 (39). Similarly, in the analysis of the prescribing antihypertensive patters in Canada between 1996 and 2006, Walker et al. found an increase in the BB monotherapy prescribing across all Canadian provinces [20]. That discrepancy may be attributed to variations in the recommendations of BB use established in each guideline. For instance, whereas the 2004 and 2006 Canadian guidelines recommended the use of BB in all hypertensive patients aged below 60 years [42,43] and the JNV 7 established BB a second line antihypertensive treatment for all patients [44], the 2004 and 2006 NICE guidelines downgraded the BB as an additional therapy for all patients [22,24]. Hence, these observations may suggest that the local guidelines markedly influence the antihypertensive prescribing patterns among general practitioners.

### Strengths and limitations

The main strength of this paper is the comparison of trends in the management of hypertension in different ethnic groups as the study area has a diverse population with a higher proportion of black patients than is the case nationally [45]. Moreover, this study showed the variation in prescribing across the different NICE patient groups over a 10 year period. Previous studies have examined the impact of clinical guidelines but few have examined the impact of a race/ethnicity treatment algorithm in a long follow-up period [33]. To our knowledge, other national interventions intend to improve antihypertensive prescribing were not launched at that time. The Quality and Outcomes Framework (QOF), a national pay for performance program, was introduced in 2004 could indirectly motivate the use of more antihypertensive medications in order to improve blood pressure control. However this financial incentive program does not have any particular recommendations on drug therapy prescription. The findings concur with variations in antihypertensive prescribing reported in the 2010 NICE implementation uptake report [35].

The use of GEE models offer also some advantages in the analysis of longitudinal data. The assessment of the interventions across groups could be more accurate because it calculates the standard errors taking into account the correlation present within repeated measures [27]. Ignoring that correlation could reduce the statistical power of the study [46]. Hence GEE models have been found more appropriate for comparing binary outcomes in longitudinal studies than the classical analysis of variance [46]. Further, these models render accurate estimators despite having data with small number of clusters [27].

The use of retrospective, routine clinical data meant that there was a lack of information about the drug selection criteria used by general practitioners in the study area. Then, we cannot provide reasons for the differences between the guideline recommendations and our reported trends. We were only able to examine the impact of the 2006 NICE guidelines over a 18 month period. However the impact is consistent to the prescribing trend observed in other surveys [35]. Additionally, the study was based on hypertensive patients registered with general practices in a specific area of London. Hence, our results may not be generalizable to other populations including black groups living in other geographical areas however the observed trends are in line with the current trends in antihypertensive prescribing reported by other authors in UK primary care [34]. Additionally, although socio-demographic characteristics could influence antihypertensive prescribing, the UK's National Health Service (NHS) aims to reduce differences in treatment among general practices across the country. UK primary care provides universal access to care to all the population. The guidelines apply to all patients regardless of socio-economic status or area of residency.

## Conclusion

Among hypertensive patients treated in primary care in Wandsworth, there was a constant increase in the use of 2006 NICE recommended antihypertensive treatment in both younger non-black patients and black patients between 2000 and 2007. The introduction of the 2006 NICE guidelines had the greatest impact on prescribing for younger non-black patients. Lower guideline-associated increases in recommended prescribing among black patients may be due to higher levels of that recommended prescribing in this patient group at baseline. The treatment offered to older non-black patients was less influenced by the 2006 NICE guidelines. Hence, the ethnic-age specific NICE guidelines influence antihypertensive prescribing but the impact of these guidelines can vary across different patient groups.

## Abbreviations

ACEI: Angiotensin converting enzyme inhibitors antihypertensive drug class; BB: Beta-blocker antihypertensive drug class; BHS: British Hypertension Society; CCB: Calcium antagonist blocker antihypertensive drug class; CI: Confidence interval; DD: Diuretic antihypertensive drug class; GEE: Generalized estimating equations; JNC 7: The Seventh Report of the Joint National Committee on Prevention, Detection, Evaluation, and Treatment of High Blood Pressure; NICE: National Institute for Health and Clinical Excellence; OR: Odds ratio; QOF: The Quality and Outcomes Framework; QIC: The independence model criterion; NHS: National Health Service; UK: The United Kingdom.

## Competing interests

The authors declare that they have no competing interests.

## Authors’ contributions

All authors participated in discussions about the analysis and have revised versions of the presented study. Dr. C M is the guarantor of the study. All authors read and approved the final manuscript.

## Pre-publication history

The pre-publication history for this paper can be accessed here:

http://www.biomedcentral.com/1472-6963/14/87/prepub

## Supplementary Material

Additional file 1 Appendix annual variation in the odds ratio of being prescribed each antihypertensive drug class by NICE patient groups between 2000 and 2007*. Click here for file
