# Supplementary figures and images for: Impact of ethnic-specific guidelines for anti-hypertensive prescribing in primary care in England: a longitudinal study
Source: BMC Health Serv Res. 2014 Feb 25;14:87. doi: 10.1186/1472-6963-14-87 (PMC3943578; doi:10.1186/1472-6963-14-87)

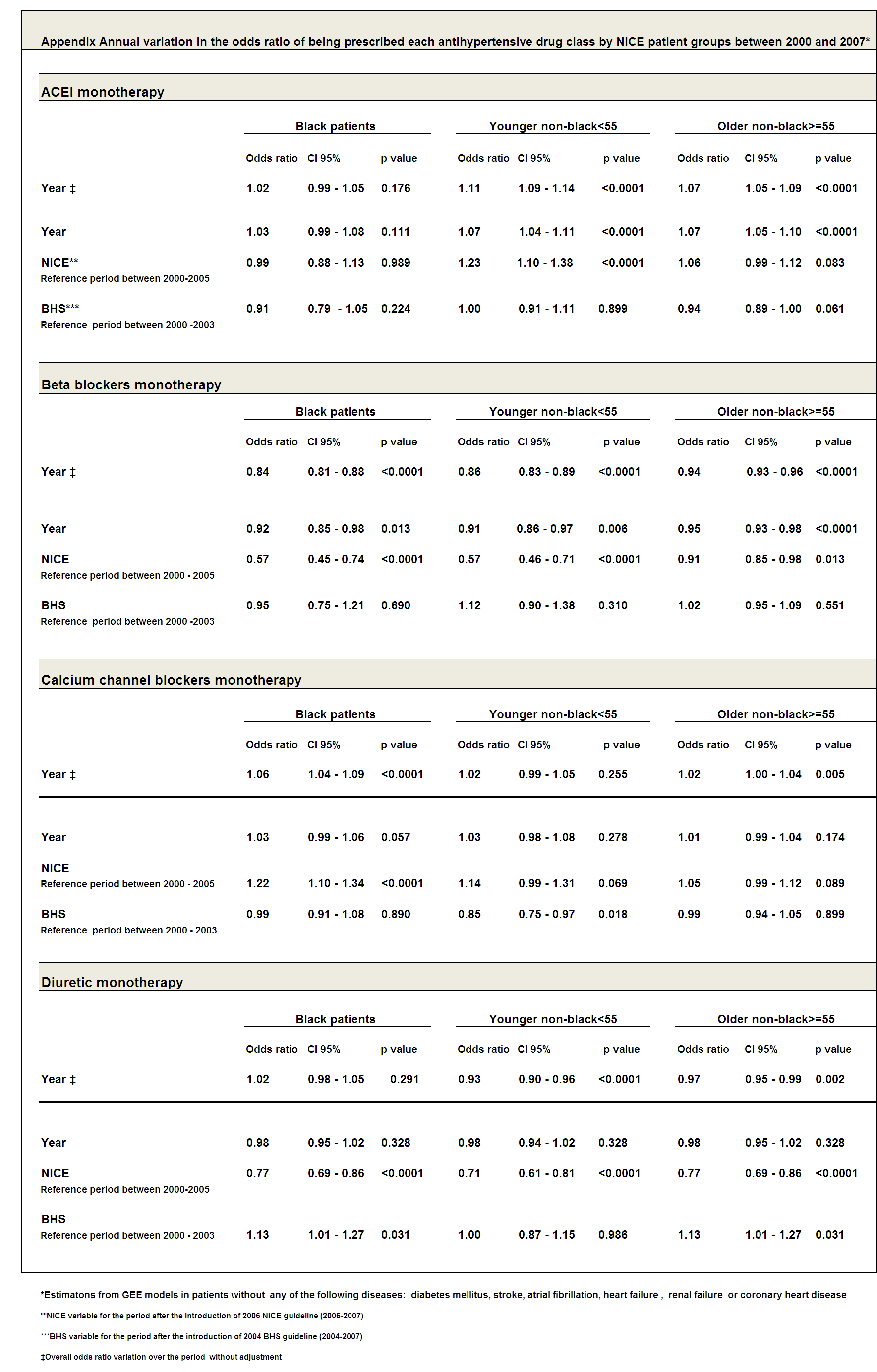

Supplement: Additional file 1 — Appendix annual variation in the odds ratio of being prescribed each antihypertensive drug class by NICE patient groups between 2000 and 2007*. [file 1472-6963-14-87-S1.docx]
